# Biomimetic 3D-Printed *Salvinia molesta* Surfaces for Geometry-Driven Wetting Control

**DOI:** 10.1021/acsami.6c06063

**Published:** 2026-06-20

**Authors:** Vegar Stubberud, Kasper E. B. Skovly, Carlos Alberto Dorao, Håkon J. D. Johnsen

**Affiliations:** † Department of Mechanical and Industrial Engineering, 570217 Norwegian University of Science and Technology, Richard Birkelands vei 2B, 7034 Trondheim, Norway; ‡ Department of Energy and Process Engineering, 8018 Norwegian University of Science and Technology, Kolbjørn Hejes vei 1B, 7034 Trondheim, Norway

**Keywords:** two-photon polymerization, Salvinia, 3D printing, Cassie−Baxter, additive manufacturing, microstructured surfaces

## Abstract

Functional bioinspired surfaces have received increasing attention due to their potential in water-repellent, drag-reducing, and antifouling applications. The Salvinia effect, inspired by the leaf of *Salvinia molesta*, provides a route for the design of water-repellent surfaces, but the complex three-dimensional trichome geometry and the combination of hydrophobic and hydrophilic regions have limited its artificial reproduction. Two-photon polymerization (2PP) offers a direct high-resolution route to fabricate such geometries and rapidly modify local features without molding or multistep lithographic processing. In this work, we demonstrate 2PP fabrication of Salvinia-inspired microstructured surfaces and use them to study geometry-driven wetting in a monomaterial system. The structures consisted of a base trichome geometry fabricated with different print profiles and two apex-modified designs used to probe geometry-driven pinning in the same printed material. The printed microstructures achieved conservative apparent static contact angles (CA) exceeding 135° and a meniscus retention distance of 0.47 mm. These results show that 2PP can decouple the apparent wetting behavior of structured surfaces from the intrinsic hydrophilicity of the printed material.

## Graphical abstract

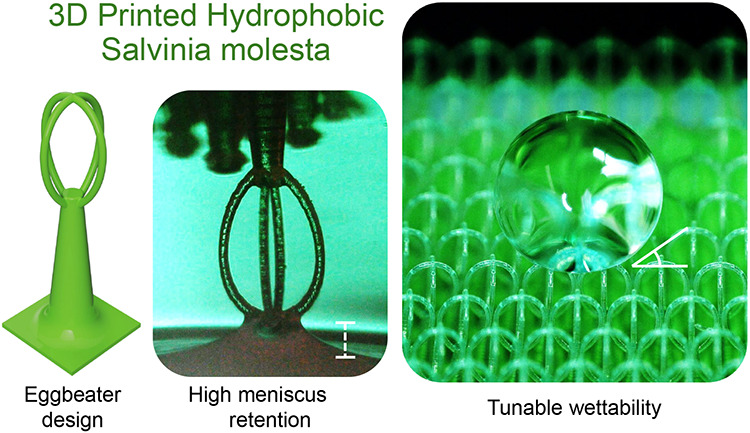

## Introduction

Natural surfaces provide useful design principles because structure, chemistry, and mechanics are often combined across multiple length scales. Artificial bioinspired surfaces therefore usually simplify the natural system while retaining the features most relevant to the target function. Bioinspired surfaces have already been applied in man-made products, e.g., gecko inspired adhesives,^1,2^ sharkskin inspired swimming apparel,^3,4^ structural colors inspired by butterflies^5,6^ and superhydrophobic surfaces inspired by the lotus plant.^7,8^ The aquatic fern *Salvinia molesta* (SM) can retain a layer of air when submerged underwater, known as the “Salvinia effect”, and acts as a superhydrophobic surface.^9–13^ These wetting properties motivate future drag-reduction and antifouling concepts for water-contacting surfaces.^14–24^ The trichome covered surface of the *S. molesta*, pictured in Figure 1, supports water droplets in a Cassie–Baxter wetting state in the natural plant.^9^

**Figure 1:**
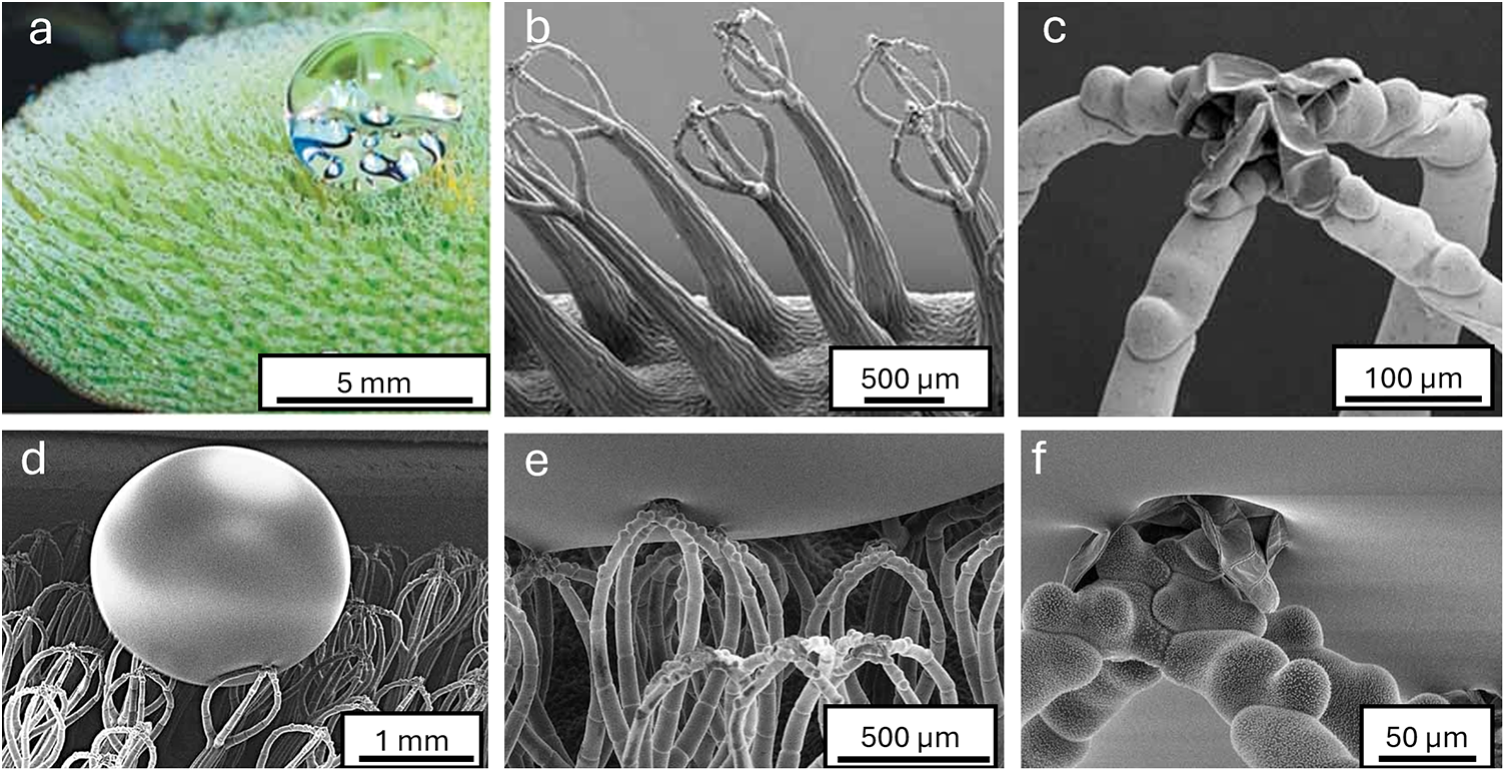
(a) The *S. molesta* leaf. (b) The trichomes on the surface of the *S. molesta*. (c) The apex of the trichomes. (d) A droplet resting on the trichomes in a Cassie–Baxter state. (e, f) Pinning of the droplet to the apex. Images (a–f) are adapted from Barthlott et al.^9^ with permission. Copyright 2010, Wiley.

The unique eggbeater-like trichome geometry, together with the combination of hydrophobic and hydrophilic regions, has made SM a prominent model for biomimetic structures. The trichomes are covered in hydrophobic wax crystals^25^ except for the apex, where a patch of dead cells has hydrophilic properties.^26^ These dead cells cover around 2% of the surface structure and pin water, acting as an anchor that stabilizes the water–air interface in the natural plant.^9^ Several efforts to replicate the properties of *S. molesta* have been made in recent years^11,27,28^ with static contact angle measurements ranging from hydrophobic to superhydrophobic.

The size and shape of the trichomes vary according to the position of the leaf^26^ with an increase in height toward the center of the leaf. This hierarchical architecture is believed to promote air trapping via a pneumatic-spring mechanism.^29^ Although the natural elasticity of the trichomes is believed to contribute to the Salvinia effect,^7^ researchers have recreated the air trapping effect with stiff bioinspired trichomes.^30,31^

Bing et al. reviews previous attempts to produce artificial *S. molesta* and a recurring limitation is the difficulty of combining complex three-dimensional geometry with spatially controlled wetting properties. Lithography, electrospinning, molding, coating-based approaches, and conventional 3D printing can be powerful, but often require simplified structures, multistep processing, or lower geometric resolution than needed for local apex modifications. This highlights that replicating hierarchically structured and chemically heterogeneous surfaces remains a challenge. In this context, additive manufacturing offers a promising route,^32–35^ and two photon polymerization (2PP) provides an opportunity for direct fabrication of complex biomimetic geometries with high resolution, rapid design iteration, and local geometric control at the trichome apex.

2PP printing is an additive manufacturing method that enables submicrometre resolution and centimeter-scale build volumes, making it suitable for fabricating SM-inspired trichomes, which are millimeter-sized structures with micrometre-scale features. The 2PP process uses femtosecond laser pulses to induce two photon absorption at the laser focal point, causing highly localized polymerization in the resin, illustrated in Figure 2b. The properties of natural *S. molesta* are the result of evolution, and the structures, with nanoscale wax crystals at the surface and a hydrophilic apex, are optimized for the specific needs of the plant. A bioinspired design therefore needs to simplify the structure while retaining features relevant to water repellence and local pinning. Using only 2PP, we are limited here to a monomaterial structure and its inherent wetting properties. Despite the potential of 2PP for designing complex microstructures, its use for tuning wetting properties and separating material effects from geometry effects remains limited. In this work, we use the 2PP printer NanoOne to manufacture bioinspired *S. molesta* structures with tunable apparent wettability. This is achieved by altering the print parameters and by introducing experimental apex designs that probe whether local geometry can promote pinning without adding chemical heterogeneity. The design, production and use of such a structure is illustrated in Figure 2. To investigate the wetting properties of the 2PP printed Salvinia-inspired structures, we first characterized the intrinsic wetting properties of the UpPhoto material under different 2PP print profiles using featureless slab samples. We then evaluated the wetting behavior of the printed microstructured surfaces through static contact angle, contact angle hysteresis (CAH), and sliding angle measurements, and quantified the ability of the structures to pin to the water surface using a meniscus retention test.

**Figure 2:**
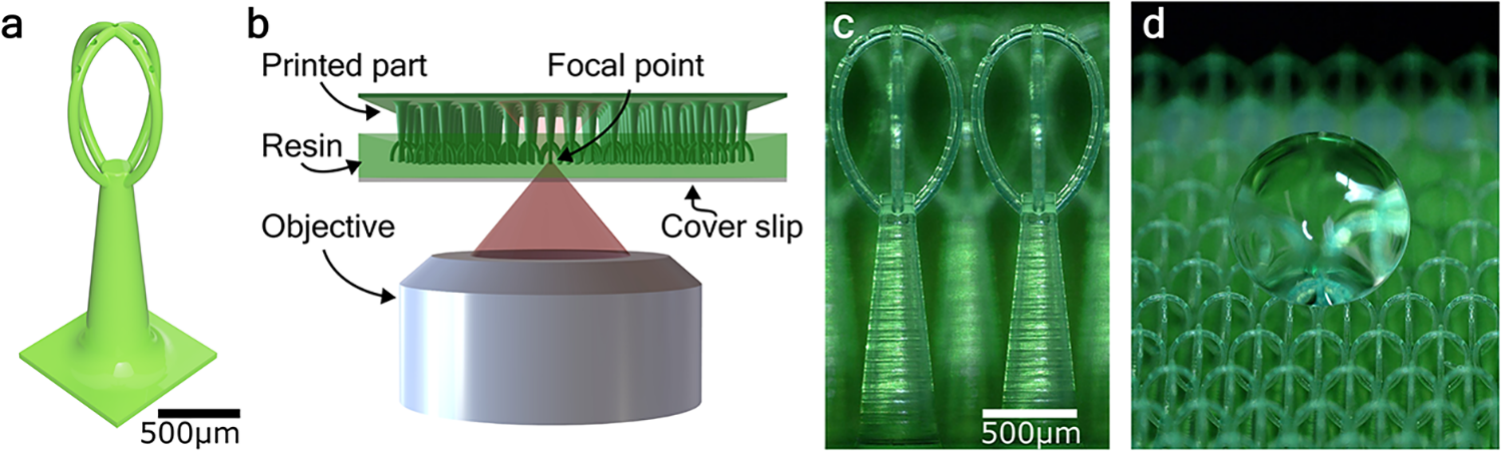
(a) 3D model of experimental trichome design. (b) Illustration of 2PP print process of the trichome array. (c) Side view of the 2PP printed trichome presented in this work. (d) Droplet resting on top of the trichome array.

## Results and Discussion

### Design of 2PP Salvinia-Inspired Structures

In this article, we present a simplified bioinspired Salvinia geometry together with two experimental apex modifications of the original bioinspired design, illustrated in Figure 3a–c. The simplified design is inspired by the tallest of the trichomes in the *S. molesta* and consists of a 1526 μm tall stem with 4 filaments forming an oval shape from the stem with a width of 704 μm, a height of 1009 μm and a diameter of 70 μm. The structures are arranged in a hexagonal pattern with a center-to-center distance of 815 μm as seen in Figure 3d,3e. All structures have the same height and orientation to further investigate the effect of print settings and experimental modifications.

**Figure 3:**
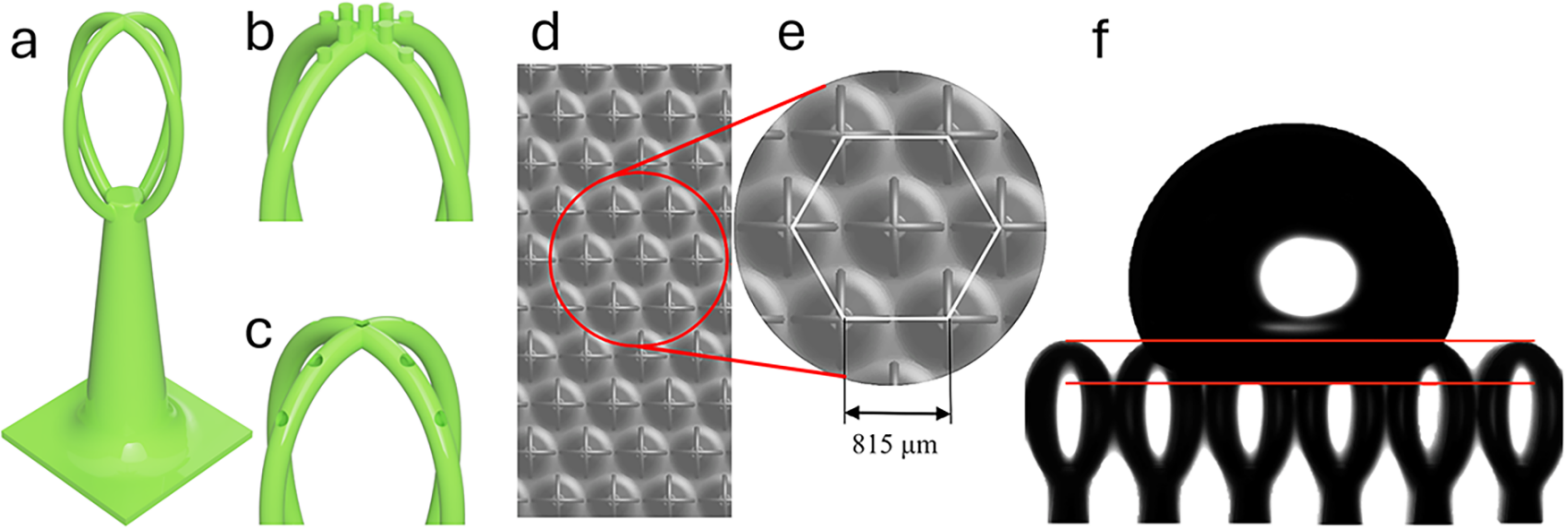
(a) Bioinspired *S. molesta* base design. (b) Modified apex of EX1. (c) Modified apex of EX2. (d, e) Top view of structure arrangement. (f) Side view of droplet resting on trichome array with lines representing potential CA measurement reference.

An observed property of the *S. molesta* is the ability of the trichomes to pin to water and retain the meniscus when pulled from the water.^29^ This effect is believed to contribute to the stability of the water–air interface together with the pneumatic spring mechanism discussed by Gandyra et al.^29^ The experimental modifications to the base structure are made to investigate how local geometry can alter apparent wettability and increase this pinning effect, rather than using coatings or other postprocessing steps. Because the structures are printed from a single material, these modifications isolate the role of local apex geometry in water pinning. Previous studies demonstrate how geometric alterations affect wetting properties, such as increasing the number of trichomes^36^ or using multi walled carbon nanotubes to increase surface roughness.^37^ Generally, increasing the surface roughness of a hydrophilic material in a Wenzel state causes more adhesion, and therefore potentially more pinning. This is the basis of experimental design 1 (EX1), where pillars of 40 μm in diameter are added to the apex of the base structure. The second experimental design (EX2) incorporates hollow hairs in the apex with holes facing away from the base to test whether local capillary geometry can promote water pinning and meniscus retention.

### Intrinsic Material Properties

The performance of the Salvinia-inspired structure depends on the intrinsic wetting properties of the selected material, both as a flat slab and with the surface quality inherent from the 2PP printing process. The laser intensity and voxel size may influence wetting because different exposure conditions can change the degree of polymer cross-linking.^38^ Mapping such properties to the print settings provides a basis for interpreting the wetting properties of the structured samples.

To investigate intrinsic material properties, cubes of 4500 μm × 4500 μm × 1500 μm were printed in VAT mode with UpPhoto material with varying settings and objectives: 5× coarse mode (5×C), 5× fine mode (5×F), 10× coarse mode (10×C) and 10× fine mode (10×F). These settings were selected to span a practical resolution-throughput trade-off within the standard NanoOne print profiles. The 10× objective has a smaller voxel size, delivering higher resolution at the cost of volumetric printing speed, while the 5× objective has lower resolution and faster volumetric printing speed. Coarse and fine modes were compared to test whether the infill profile affects surface wetting and manufacturability for the same nominal geometry. All cubes were printed with the default field of view (FOV) settings (2828 μm by 2828 μm for the 5× and 1414 μm by 1414 μm for the 10×). As the samples are printed on a glass substrate, the side facing the substrate will be smooth and flat, while the top side will have an inherent surface structure from the 2PP printing process. The advancing contact angle (ACA) and the receding contact angle (RCA) were measured with a tensiometer for the flat side of the prints, and the static CA was measured for both sides of all prints.

The static contact angles of the flat side of all samples ranged between 63.3° ± 2.1° and 68.4° ± 0.8°, indicating that the surface of the material is hydrophilic. For the upper side with an inherent surface structure from the 2PP process, the static contact angle was consistently higher, ranging from 69.5° ± 0.1° to 76.6° ± 0.7°. This is consistent with the surface texture produced by the 2PP printing process influencing wettability. The CAs for both sides are presented in Figure 4.

**Figure 4:**
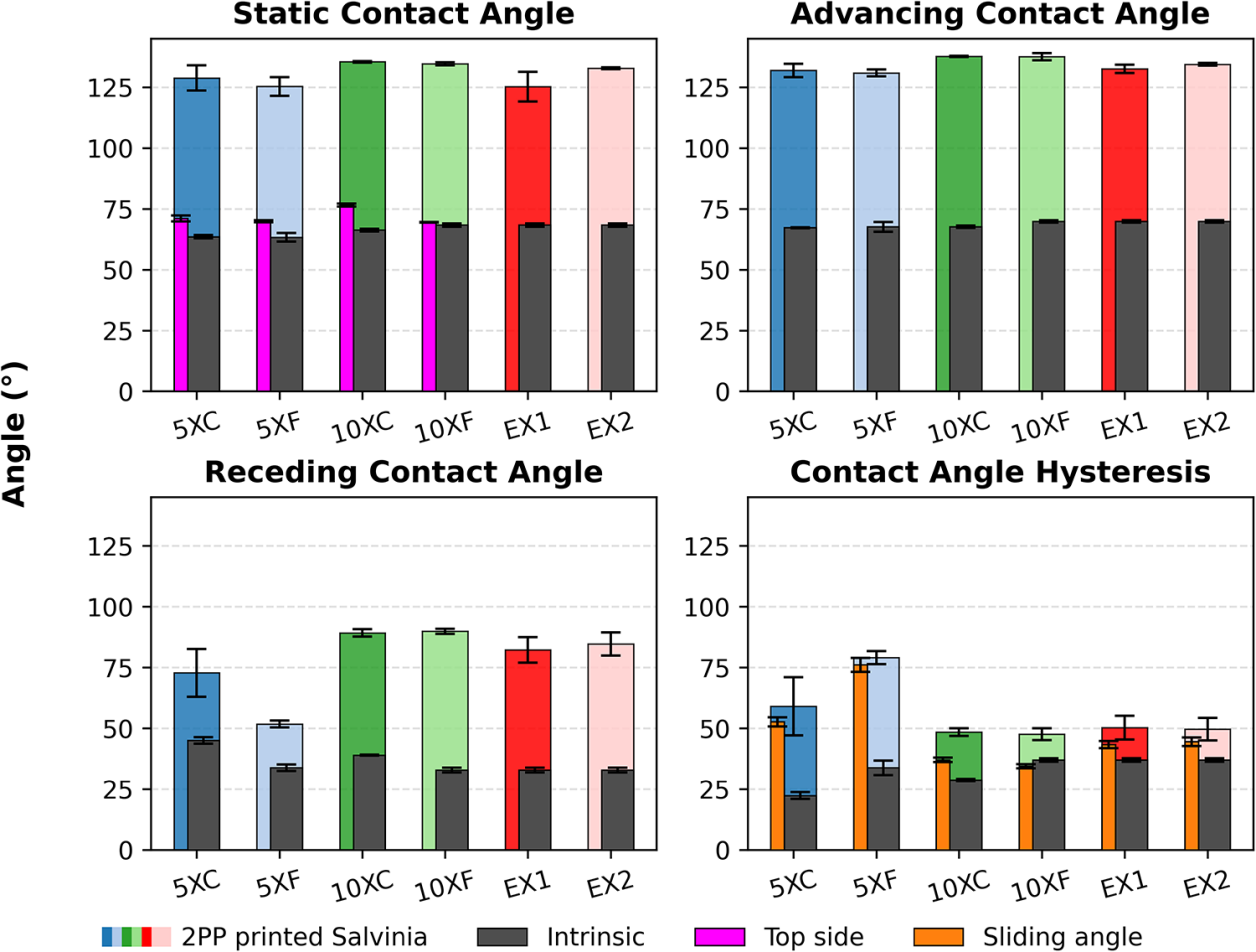
Static contact angle (CA), advancing CA (ACA), receding CA (RCA), contact angle hysteresis (CAH), and sliding angle (SA). Gray and magenta bars represent intrinsic measurements on the flat bottom side and printed top side of the slabs, respectively. Orange bars represent sliding angles, and the wide colored bars represent the apparent wetting properties of the 2PP printed Salvinia-inspired samples.

Interestingly, when the CAH was measured on the flat side, the infill mode appeared to affect the adhesion properties of the material, despite having little impact on the static contact angle. Using fine mode, the material appeared to be more adhesive in contact with water. The CAH was also higher for the higher-magnification objective. As a result, the highest CAH was found for the printing configuration with the highest resolution, 10×F (CAH of 37.0° ± 0.7°), followed by 5×F (CAH of 33.8° ± 2.9°). The lower-CAH slabs measured a CAH of 28.7° ± 0.5° for the 10×C configuration and 22.3° ± 1.4° for the 5×C configuration. These measurements were taken using the needle-in-the-sessile-droplet method proposed by Korhonen et al.^39^ The ACA, RCA and CAH are presented in Figure 4. CAH was not measured on the top side as the stitch lines between the FOVs interfered with the droplet.

The print settings influence the wetting properties of the printed cube. With different printing profiles, the voxel size, scanning speed, and laser intensity change. These changes could alter cross-linking or other near-surface material properties,^38^ and the present measurements do not separate these effects from profile-dependent surface texture. For the top side, the inherent 2PP surface structure will also change depending on the printing profile and affect wettability. As the intended Salvinia-inspired geometry differs greatly from the test slabs, the question of which print setting is most suitable remains linked to both geometry fidelity and wetting performance.

### Contact Angle Measurements of Salvinia-Inspired Structures

Contact angle is traditionally measured from the surface plane, as that is where the droplet rests. In this case, the bottom plane of the droplets is not defined, somewhere between the red lines in Figure 3f. This nonplanar trichome array makes the droplet baseline ambiguous and limits direct comparison with standard flat-surface contact-angle values. The measured CA in this article is therefore based on the top red line, as this plane is the same for all structures. This gives a consistent and conservative apparent contact angle compared to a lower reference line. No lower-baseline contact-angle range is reported because the corresponding reference plane was not measured consistently across all samples.

Because intrinsic material measurements display varying wetting properties for each print profile, the base structure is printed with the exact same profiles to investigate how the profiles affect wetting in a more complex geometry. As both experimental designs have a finer feature size, they are only printed with the 10×F profile to ensure that all features are printed. The sessile droplet method indicates that the bioinspired surfaces produced water-repellent structured states, despite the hydrophilic material properties. The surfaces printed with the 10× objective had the highest conservative apparent CA values, with a CA of 135.4° ± 0.3° with fine mode and 134.6° ± 0.7° with coarse mode. The 5× surfaces had lower apparent CA values, with the measured CA for the 5×C being 128.8° ± 5.2° and 125.3° ± 3.8° for 5×F. This trend agrees with the initial material characterization, as the apparent hydrophobicity increases with the magnification of the objective, while only minor differences were observed between different infill modes. The contact-angle data are consistent with droplets resting partly on the printed structures and spanning air-filled gaps between them, indicating Cassie–Baxter-like apparent wetting under sessile-droplet conditions. For hysteresis and sliding angle, the 5×F sample is notable for an increased hysteresis and a sliding angle of 76.1° ± 2.9°, considerably higher than that of the 5×C (SA of 52.7° ± 1.9°). The cause of the increase in SA of 5×F may be the local apex geometry rather than the nominal print profile alone. Visual inspection of the 5×F structures reveals a slight change in filament geometry near the apex with taller and more oval structures, as seen in Figure 5b. This is likely a print defect, and we therefore treat the strong pinning-related performance of 5×F as potentially influenced by this unintended local geometry. All bioinspired samples are shown in Figure 5. The high hysteresis and sliding angle nevertheless suggest that small changes in apex geometry can strongly affect water pinning. Quantitative apex profilometry or higher-magnification SEM would be needed to separate the effects of nominal print profile and unintended local geometry.

**Figure 5:**
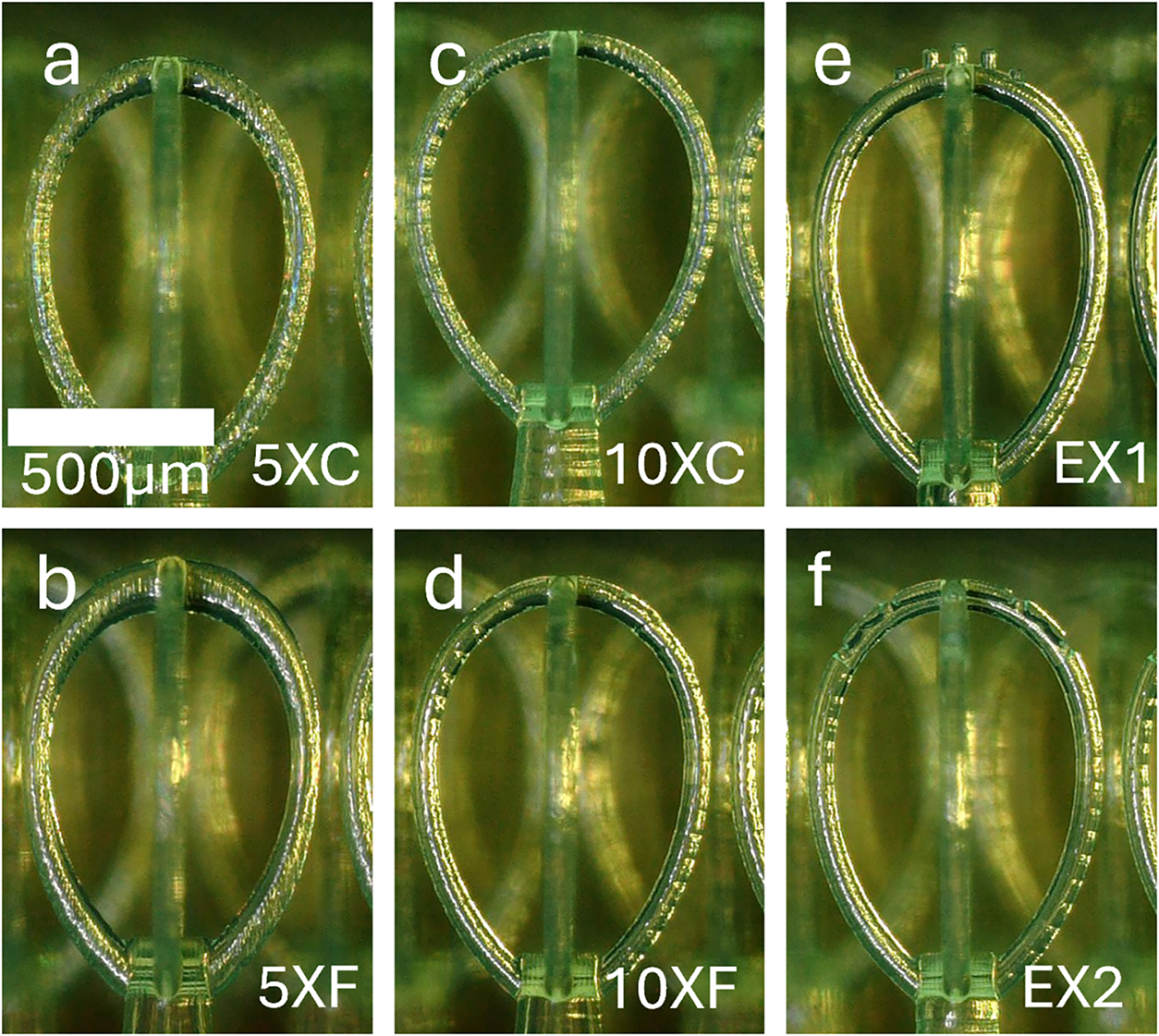
Side view of 5×C (a), 5×F (b), 10×C (c), 10×F (d), EX1 (e) and EX2 (f).

The infill mode did not seem to strongly affect the surfaces printed with the 10× objective, as similar results were found between the two variations (SA of 37.0° ± 0.9° for 10×C and 34.5° ± 0.8° for 10×F).

Despite the design changes for EX1 and EX2, the CAH did not change substantially due to the new tip geometry. However, static CA was reduced by approximately 10° by adding pillars to the tips compared to the other surfaces printed with the same magnification (from 135.4° ± 0.3° to 125.2° ± 6.1°). Because EX1 and EX2 are printed from the same material, the change is attributed to local geometry rather than a change in surface chemistry.

Instead, the result suggests that local geometric changes at the apex can alter apparent wetting and pinning. The surface with hollow filaments performed similarly to the unmodified structures in both measurements.

### Meniscus Retention

The meniscus retention distance is a measurement of the ability of a trichome to hold to the surface of the water while being pulled up. Barthlott et al. measured this distance using a real SM trichome and compared it to a Teflon coated trichome. The untreated version measured a meniscus retention distance of 0.2 mm, which was twice as much as for the Teflon coated sample.^9^ In the present system, the largest measured retention distance was 0.47 mm, although this comparison should be made cautiously because the artificial trichomes differ in geometry, material, and test conditions from the natural plant. The experimental setup and the result from the meniscus retention test are shown in Figure 6. The strongest pinning (0.47 mm ± 0.05 mm) was found for the structure printed with 5×F and was also the most adhesive and least hydrophobic among the different samples. As discussed above, the 5×F response may be influenced by unintended local apex geometry. The capillary effect-inspired trichome had a similar retention distance, despite being more hydrophobic and less adhesive, with a retention distance of 0.46 mm ± 0.01 mm. This suggests that microscale channels can increase the pinning effect with minimal effects on the static CA and CAH. Although the addition of pillars reduced static CA, it did not measurably increase pinning under the present test conditions. The results were similar to those of the unmodified trichome printed with the same configuration (0.42 mm ± 0.01 mm).

**Figure 6:**
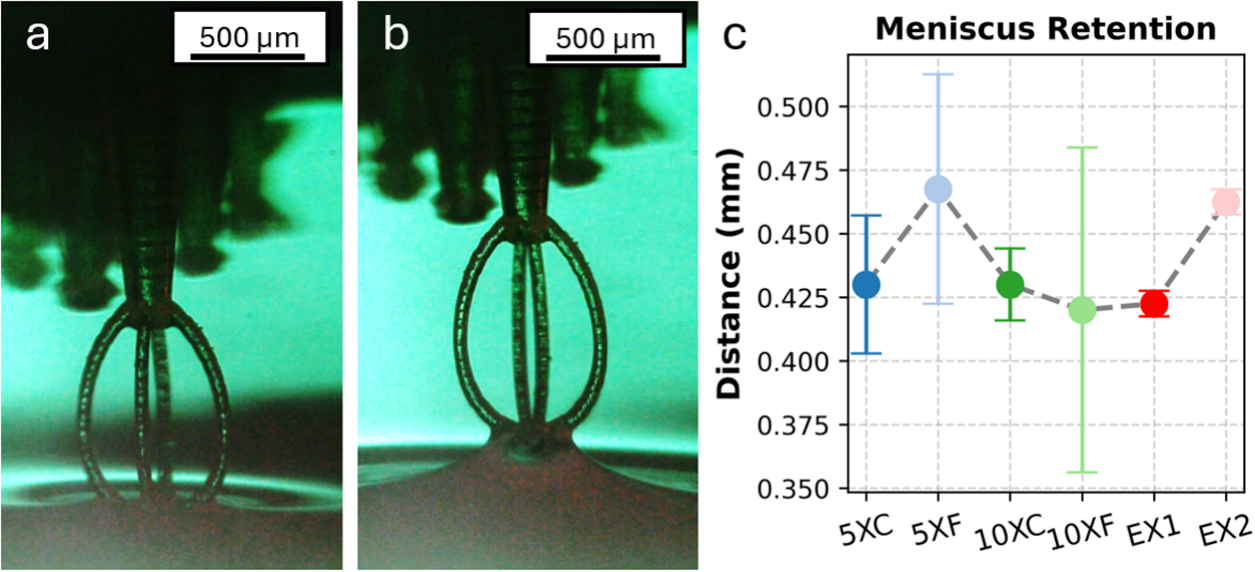
Side view of the meniscus retention measurement (a, b). Results from meniscus retention test for all bioinspired samples (c).

## Conclusion

In this work, we demonstrate the use of two photon polymerization for 3D printing functional surfaces inspired by the *S. molesta*. Even with an intrinsically hydrophilic material, the printed trichome arrays produced Cassie–Baxter-like apparent wetting, with conservative apparent contact angle measurements exceeding 135° and varying wetting properties depending on print profile. The structures also retained the meniscus when pulled from water, with a maximum measured retention distance of 0.47 mm. Further work should evaluate underwater air-layer stability and long-term air retention to determine how closely these monomaterial structures reproduce persistent Salvinia-effect air retention. Two experimental designs were presented, one with a hollow apex and one with added pillars on the apex surface. Together, these designs show how apex geometry can be varied within a monomaterial print to tune pinning behavior. The hollow apex achieved higher meniscus retention without strongly altering the contact angle measurements, demonstrating geometric tuning of the wetting properties. The print profile that stood out the most was the 5×F with the highest hysteresis, sliding angle, and meniscus retention. Visually, this print also differs geometrically from the rest, with a thicker and more oval filament shape near the apex. While likely being a print defect, it supports the conclusion that local apex geometry can strongly affect wetting and pinning, and this effect should be quantified in future work. As this study demonstrates, two photon polymerization is a useful tool for producing and prototyping bioinspired geometries and for studying geometry-driven wetting behavior in a monomaterial system. Different resins or coatings would be expected to shift the intrinsic material contribution, while the present monomaterial approach isolates the geometric contribution more directly. Such design freedom may support future studies of water-repellent, antifouling, drag-reducing, or biomedical microstructured surfaces, but those application-level functions were not directly tested here.

## Experimental Section

### Fabrication

The prints were made with the UpNano NanoOne 1000 using the UpPhoto material in VAT mode with standard glass substrates. Standard printing profiles for coarse and fine mode were used for the 5× and 10× objectives. These objective and infill combinations were chosen to compare higher-throughput printing with higher-resolution printing while retaining the same nominal structure. The prints were developed in isopropanol to remove the unpolymerized resin before testing. For the prints in fine mode, the model was sliced into two parts: the base with the stem and the trichome. The base and stem were printed in coarse mode to decrease the print time. The print times were around 30 min, 4, 5, and 24 h for 5×C, 5×F, 10×C, and 10×F respectively and around 9 h for both EX1 and EX2. These print times illustrate the practical resolution-throughput trade-off of the selected settings rather than a demonstrated route to large-scale manufacturing. No independent dimensional error range was measured for the printed trichomes.

### Contact Angle Measurements

The OneAttension Theta Flex optical tensiometer was used to perform contact angle measurements. The sample was placed in a self-made humidity chamber to achieve stable static CA measurements. The static CA values were based on the average CA of a 5 s interval, 10 s after the release of the droplet. Advancing and receding contact angles were measured using the sessile drop method for hysteresis measurements on the smooth side of the cubes using a dispensing rate of 0.05 μL/s. For the bioinspired structures, the advancing and receding contact angles were measured using a tilted plate. A 30 μL droplet was placed on the surface using a Thermo Scientific Finnpipette F2. The tensiometer was then tilted at 0.5°/s until the droplet rolled. The contact angles were recorded with the camera on the tensiometer. For the structured samples, contact angles were measured using the upper common reference line of the trichome array to avoid sample-dependent baseline selection. All measurements were repeated four times for each sample and are reported as mean ± standard deviation (SD) unless otherwise noted. No formal statistical significance testing was performed; differences are therefore interpreted descriptively, with emphasis on larger trends rather than small numerical differences.

### Meniscus Retention Measurements

All except one structure of the bioinspired samples were removed using a razor blade. The sample was mounted onto a motorized stage facing down into the humidity chamber. The sample was lowered into the water and lifted with increments of 0.01 mm until release. The height difference was noted, and the measurements were repeated four times for each sample. All measurements are reported as mean ± standard deviation (SD) unless otherwise noted. No formal statistical significance testing was performed for the meniscus retention measurements.

## References

[1] Boesel L. F., Greiner C., Arzt E., del Campo A. (2010). Gecko-Inspired
Surfaces: A Path to Strong and Reversible Dry Adhesives. Adv. Mater..

[2] Sikdar S., Rahman M. H., Siddaiah A., Menezes P. L. (2022). Gecko-Inspired Adhesive
Mechanisms and Adhesives for Robots—A Review. Robotics.

[3] Oeffner J., Lauder G. V. (2012). The hydrodynamic function of shark
skin and two biomimetic
applications. J. Exp. Biol..

[4] Chen D., Liu Y., Chen H., Zhang D. (2018). Bio-inspired drag reduction surface
from sharkskin. Biosurf. Biotribol..

[5] Tadepalli S., Slocik J. M., Gupta M. K., Naik R. R., Singamaneni S. (2017). Bio-Optics
and Bio-Inspired Optical Materials. Chem. Rev..

[6] Liu H., Li M., Huang G., Li J., Xu F. (2021). Bioinspired Microstructure
Platform for Modular Cell-Laden Microgel Fabrication. Macromol. Biosci..

[7] Solga A., Cerman Z., Striffler B. F., Spaeth M., Barthlott W. (2007). The dream
of staying clean: Lotus and biomimetic surfaces. Bioinspiration Biomimetics.

[8] Wang S., Liu K., Yao X., Jiang L. (2015). Bioinspired
Surfaces with Superwettability:
New Insight on Theory, Design, and Applications. Chem. Rev..

[9] Barthlott W., Schimmel T., Wiersch S., Koch K., Brede M., Barczewski M., Walheim S., Weis A., Kaltenmaier A., Leder A., Bohn H. F. (2010). The Salvinia Paradox: Superhydrophobic
Surfaces with Hydrophilic Pins for Air Retention Under Water. Adv. Mater..

[10] Koch K., Bohn H. F., Barthlott W. (2009). Hierarchically Sculptured Plant Surfaces
and Superhydrophobicity. Langmuir.

[11] Bing W., Wang H., Tian L., Zhao J., Jin H., Du W., Ren L. (2021). Small Structure,
Large Effect: Functional Surfaces
Inspired by Salvinia Leaves. Small Struct..

[12] Wang G., Guo Z., Liu W. (2014). Interfacial Effects
of Superhydrophobic Plant Surfaces:
A Review. J. Bionic Eng..

[13] Cerman, Z. ; Striffler, B. F. ; Barthlott, W. Functional Surfaces in Biology: Little Structures with Big Effects; Gorb, S. N. , Ed.; Springer Netherlands: Dordrecht, 2009; Vol. 1, pp 97–111.

[14] Weisheit J., Schneider V. E., Serr J. M., Hagemeister N., Oeffner J. (2021). Validation of a Flow Channel to Investigate Velocity
Profiles of Friction-Reducing Ship Coatings. TransNav, Int. J. Mar. Navig. Saf. Sea Transp..

[15] Oeffner, J. ; Jalkanen, J.-P. ; Walheim, S. ; Schimmel, T. In From Nature to Green Shipping: Assessing the Economic and Environmental Potential of AIRCOAT on Low-Draught Ships; Proceedings of 8th Transport Research Arena TRA 2020, April 27-30, 2020, Helsinki, Finland, 2020.

[16] Kavalenka M. N., Vüllers F., Lischker S., Zeiger C., Hopf A., Röhrig M., Rapp B. E., Worgull M., Hölscher H. (2015). Bioinspired
Air-Retaining Nanofur for Drag Reduction. ACS
Appl. Mater. Interfaces.

[17] Dong H., Cheng M., Zhang Y., Wei H., Shi F. (2013). Extraordinary
drag-reducing effect of a superhydrophobic coating on a macroscopic
model ship at high speed. J. Mater. Chem. A.

[18] McHale G., Newton M. I., Shirtcliffe N. J. (2010). Immersed
superhydrophobic surfaces:
Gas exchange, slip and drag reduction properties. Soft Matter.

[19] Shirtcliffe N. J., McHale G., Newton M. I., Zhang Y. (2009). Superhydrophobic Copper
Tubes with Possible Flow Enhancement and Drag Reduction. ACS Appl. Mater. Interfaces.

[20] Balasubramanian A. K., Miller A. C., Rediniotis O. K. (2004). Microstructured
Hydrophobic Skin
for Hydrodynamic Drag Reduction. AIAA J..

[21] Ou J., Perot B., Rothstein J. P. (2004). Laminar
drag reduction in microchannels
using ultrahydrophobic surfaces. Phys. Fluids.

[22] Wang H., Fu H., Tian L., Bing W. (2024). Salvinia-inspired biomimetic antifouling
film with bubble shielding function. Prog. Org.
Coat..

[23] Wang D., Sun Q., Hokkanen M. J., Zhang C., Lin F.-Y., Liu Q., Zhu S.-P., Zhou T., Chang Q., He B., Zhou Q., Chen L., Wang Z., Ras R. H. A., Deng X. (2020). Design of
robust superhydrophobic surfaces. Nature.

[24] Sun H., Zhu Y., Bing W. (2025). Research on
underwater air layer retention and antifouling
ability of Salvinia-inspired biomimetic materials. Colloids Surf., B.

[25] Barthlott W., Mail M., Bhushan B., Koch K. (2017). Plant Surfaces: Structures
and Functions for Biomimetic Innovations. Nano-Micro
Lett..

[26] Barthlott W., Wiersch S., Čolić Z., Koch K. (2009). Classification of trichome
types within species of the water fern Salvinia, and ontogeny of the
egg-beater trichomes. Botany.

[27] Kim M., Yoo S., Jeong H. E., Kwak M. K. (2022). Fabrication of Salvinia-inspired
surfaces for hydrodynamic drag reduction by capillary-force-induced
clustering. Nat. Commun..

[28] Zhang Y., Hu Y., Xu B., Fan J., Zhu S., Song Y., Cui Z., Wu H., Yang Y., Zhu W., Wang F., Li J., Wu D., Chu J., Jiang L. (2022). Robust Underwater Air
Layer Retention and Restoration on Salvinia-Inspired Self-Grown Heterogeneous
Architectures. ACS Nano.

[29] Gandyra D., Walheim S., Gorb S., Ditsche P., Barthlott W., Schimmel T. (2020). Air Retention under
Water by the Floating Fern Salvinia:
The Crucial Role of a Trapped Air Layer as a Pneumatic Spring. Small.

[30] Zhang Q., Rawal G., Qian J., Zou S., Gillingham J., Zhang J., Dong L., Lu M. (2025). Hybrid Metasurface
for On-Chip Enrichment and Quantification of Biological Samples. ACS Appl. Mater. Interfaces.

[31] Tricinci O., Terencio T., Mazzolai B., Pugno N. M., Greco F., Mattoli V. (2015). 3D Micropatterned Surface
Inspired by *S. molesta* via Direct Laser
Lithography. ACS Appl. Mater. Interfaces.

[32] Xiang Y., Huang S., Huang T.-Y., Dong A., Cao D., Li H., Xue Y., Lv P., Duan H. (2020). Superrepellency of
underwater hierarchical structures on Salvinia leaf. Proc. Natl. Acad. Sci. U.S.A..

[33] Bunea A.-I., Szczotka N., Navne J., Taboryski R. (2023). Single-step
fabrication of superhydrophobic surfaces by two-photon polymerization
micro 3D printing. Micro Nano Eng..

[34] Yang Y., Li X., Zheng X., Chen Z., Zhou Q., Chen Y. (2018). 3D-Printed
Biomimetic Super-Hydrophobic Structure for Microdroplet Manipulation
and Oil/Water Separation. Adv. Mater..

[35] He, Q. ; Bethers, B. ; Tran, B. ; Yang, Y. In 3D Printing of Salvinia Water Fern-Inspired Superhydrophobic Structures; ASME 2022 17th International Manufacturing Science and Engineering Conference, ASME, 2022.

[36] Tricinci O., Terencio T., Pugno N. M., Greco F., Mazzolai B., Mattoli V. (2017). Air Trapping Mechanism in Artificial Salvinia-Like
Micro-Hairs Fabricated via Direct Laser Lithography. Micromachines.

[37] Tang T., Dwarampudi G. S. K. A. R., Li X. (2023). Electrically Assisted
Vat Photopolymerization of Bioinspired Hierarchical Structures with
Controllable Roughness for Hydrophobicity Enhancement Using Photocurable
Resin/Carbon Nanotube. JOM.

[38] Fu H., Yu B. (2024). 3D micro/nano hydrogel
structures fabricated by two-photon polymerization
for biomedical applications. Front. Bioeng.
Biotechnol..

[39] Korhonen J. T., Huhtamäki T., Ikkala O., Ras R. H. A. (2013). Reliable Measurement
of the Receding Contact Angle. Langmuir.

